# Interfering with ROS Metabolism in Cancer Cells: The Potential Role of Quercetin

**DOI:** 10.3390/cancers2021288

**Published:** 2010-06-14

**Authors:** Lara Gibellini, Marcello Pinti, Milena Nasi, Sara De Biasi, Erika Roat, Linda Bertoncelli, Andrea Cossarizza

**Affiliations:** Department of Biomedical Sciences, School of Medicine, University of Modena and Reggio Emilia, Via Campi 287, 41125 Modena, Italy; E-Mails: lara.gibellini@unimore.it (L.G.); mpinti@unimore.it (M.P.); mnasi@unimo.it (M.N.); debiasisara@yahoo.it (S.D.B.); erikaroat@gmail.com (E.R.); linda.bertoncelli@unimore.it (L.B.)

**Keywords:** cancer, reactive oxygen species, redox homeostasis, flavonoids, quercetin

## Abstract

A main feature of cancer cells, when compared to normal ones, is a persistent pro-oxidative state that leads to an intrinsic oxidative stress. Cancer cells have higher levels of reactive oxygen species (ROS) than normal cells, and ROS are, in turn, responsible for the maintenance of the cancer phenotype. Persistent ROS stress may induce adaptive stress responses, enabling cancer cells to survive with high levels of ROS and maintain cellular viability. However, excessive ROS levels render cancer cells highly susceptible to quercetin, one of the main dietary flavonoids. Quercetin depletes intracellular glutathione and increases intracellular ROS to a level that can cause cell death.

## 1. Introduction

The role of reactive oxygen species (ROS) in carcinogenesis has been extensively investigated in several cellular and animal models, and various connections have been established. On the one hand, appropriate levels of ROS play an important role in the modulation of several physiologic responses, as ROS are part of a signaling network regulating cell function. On the other hand, excessive intracellular levels of ROS, as well as a defective antioxidant system, can give rise to pathological conditions, and even encourage the progression of these conditions [1]. The mitochondrial respiratory chain is one of the major sources of endogenous ROS, together with other oxidative enzymes, such as plasma membrane oxidases [2]. 

An abnormal regulation of ROS has a role in pathological conditions, including inflammation, atherosclerosis, angiogenesis, aging, and cancer. Cells have evolved several antioxidant defenses, including repair and detoxifying enzymes, and small scavenger molecules, such as glutathione. The intracellular ROS-scavenging system includes superoxide dismutases (SOD), glutathione peroxidase (GPx), peroxiredoxins (PRDXs), glutaredoxins, thioredoxins (TRXs), and catalases. In mitochondria, superoxide anion (O_2_^−^) can be dismutated to hydrogen peroxide (H_2_O_2_) by two enzymes, namely copper-zinc superoxide dismutase (CuZnSOD) and manganese superoxide dismutase (MnSOD), that are present in the mitochondrial matrix and in the intermembrane space, respectively. Once generated, H_2_O_2_ can be quenched by GPx in mitochondria, or by catalase in the cytosol. 

Glutathione is the major non-enzymatic component of intracellular antioxidant defenses. It is present at millimolar concentrations, and it works either as a nucleophile for efficient detoxification of reactive electrophiles, or as an antioxidant [3]. Glutathione is present in a reduced, biologically active form (hereafter referred to as GSH), and in an oxidized form, namely glutathione disulfide (GSSG) [4]. The glutathione redox system is balanced when the GSH-to-GSSG ratio is high. Almost 90% of the total GSH is maintained in the reduced form through *de novo* GSH synthesis, enzymatic reduction of GSSG by GSSG reductase (GRed), and uptake of exogenous GSH [4]. The GSH system acts together with the thioredoxin system to maintain an appropriate intracellular redox homeostasis. The thioredoxin family includes both thioredoxins and glutaredoxins, as they share the common putative fold, in which the *N*-terminal cysteine of the catalytic site is positioned near the second cysteine residue. This structure allows direct access to the disulfide bonds of the target proteins, thus favoring the proteins’ reversible reduction and participation in detoxification [5].

## 2. ROS Balance in Cancer Cells

One of the main features of cancer cells, when compared to the normal ones, is a persistent pro-oxidative state that can lead to intrinsic oxidative stress [6,7]. The enhanced ROS generation is described in several *in vitro* and *in vivo* cancer models, and strongly supports this condition. For instance, cell lines from melanoma, colon, and pancreatic carcinoma, breast and ovarian cancer, and neuroblastoma produce more H_2_O_2_ than normal, non-transformed cells [6]. Likewise, chronic lymphocytic leukemia cells obtained from patients showed an increased ROS production when compared to normal lymphocytes [8]. Multiple factors support the maintenance of a pro-oxidative cancer phenotype, such as alterations in metabolic activity, the oncogenic transformation, and when present, the loss of functional p53 [9]. 

Cancer cells show increased metabolic activity as they require high levels of energy, nucleotides, lipids, and amino acids to maintain a high rate of cell growth and proliferation. In the presence high energy demand, a shift in cell metabolism is needed to enhance oxidative phosphorylation and to promote glycolysis. This shift could assure the survival of cancer cells, as well as their propagation [10]. Glycolysis can produce ATP at a higher rate, but at a lower yield, than oxidative phosphorylation can; this may selectively advantage cancer cells when competing for energy resources [11]. Indeed, the level of the H^+^ ATP synthase β-subunit (β-F1-ATPase) is significantly reduced in tumors when compared to synthase levels in normal tissues [12], and the rates of glucose uptake are increased [13].

Other than increased aerobic glycolysis, cancer cells also utilize glucose under hypoxic or anoxic conditions, or both, through the stabilization of transcription factors, which are named hypoxia inducible factors (HIFs). HIFs regulate many pathways affecting cancer progression. Among these pathways, one of the most important is the metabolic adaptation for when the tumor microenvironment is deprived of oxygen in a total or partial manner. When oxygen is present at extremely low levels, HIFs stabilize and bind to specific hypoxia-responsive elements (HRE) on the promoter of several genes that modulate glucose transport, including GLUT1 and GLUT3, and metabolism, such as pyruvate dehydrogenase kinase 1 and hexokinase 2 [14]. As a consequence of these adaptive mechanisms, more ROS can be produced that activate HIFs pathways and that are involved in cancer initiation and growth [15]. 

The association between oncogenic activation and increased ROS levels has been well investigated. For instance, the transformation of various hematopoietic cell lines with BCR/ABL results in an increase in ROS levels compared with that of quiescent, untransformed cells [16]. Mutations that activate c-myc can generate enough ROS to damage DNA [17]. Similarly, a constitutive production of O_2_^−^ characterizes NIH3T3 cells that are transformed by overexpression of oncogenic Ras and depletion of H_2_O_2_, which derives from O_2_^−^ and inhibits the growth of Ras-transformed cells [18]. A possible connection between Ras transformation and ROS is represented by NOX1, which generates O_2_^−^ from molecular oxygen [2]. The transformation of NRK cells by KrasVal12 upregulates transcription of NOX1 and introduction of NOX1 siRNA into K-RasVal12-transformed NRK cells blocks their anchorage-independent growth and induces morphological reversion [19]. Similarly, ROS derived from NOX4 are involved in pancreatic cancer and in melanoma, whereas ROS are generated by NOX5 in esophageal adenocarcinoma cells [20,21,22].

ROS unbalance and metabolic changes could also be p53-related. p53 is one of the major tumor-suppressor genes with multiple functions in regulating genomic stability, metabolism, anti-oxidant defense, proliferation, autophagy and cell death [23]. Several studies indicate that p53 impacts ROS levels. Under normal physiologic conditions, p53 can upregulate several antioxidant genes, such as GPx, MnSOD2, the tumor protein p53-inducible nuclear protein 1 (TP53INP1), Tp53-induced glycolysis and apoptosis regulator (TIGAR), and the sestrins, SESN1 and SESN2, which encode antioxidant modulators of PRDXs [24,25,26]. In p53-deficient cancer cells, the lack of p53-dependent antioxidant modulation can increase the redox stress within the cell, allowing ROS accumulation.

## 3. Cancer Cells Adapt Unbalanced ROS Levels

Cancer cells have evolved mechanisms to protect themselves from intrinsic oxidative stress and have developed a sophisticated adaptation system that essentially involves the rearrangement of the antioxidant functions and the upregulation of pro-survival molecules [27]. Recent studies demonstrate that the transcription factor FoxM1 coordinates the negative regulation of intracellular ROS levels by stimulating the expression of detoxifying enzymes, including catalase, MnSOD, and PRDX3 [28]. Together with FoxM1, multiple pathways activating redox-sensitive transcription factors, including NFκB, HIF-1, and c-jun, may contribute to cancer cell adaptation to persistent oxidative stress. BRCA1 can stimulate antioxidant gene expression and protect cells against oxidative stress. BRCA1 regulates intracellular ROS levels in several breast carcinoma cell lines and in a non-tumor human mammary epithelial cell line [29,30]. Likewise, BRCA1-deficient mice are more sensitive to oxidant-induced mortality [31]. Mitochondrial sirtuin (SIRT)-3 induces FOXO3a translocation to the nucleus and augments FOXO3a-dependent antioxidant defense mechanisms, including via the upregulation of MnSOD and catalase [32]. The loss of SIRT3 leads to increased FOXO3a phosphorylation, inducing its nuclear export and abolishing its antioxidant response, thereby steering cells to transformation. A complete transformation is reached when the loss of SIRT3 is accompanied by alterations in oncogenes, including c-myc or Ras [32].

Higher levels of antioxidant enzymes are present in malignant tissues compared with the normal counterparts’. Increased MnSOD expression has been observed in human ovarian cancer cells and in the 7,12-dimethylbenz[a]antracene-induced ovarian cancer animal model [33,34]. Higher levels of MnSOD protein have been found in tissue from gastric and esophageal carcinomas when compared with the corresponding normal mucosa cells’, and increased expression of both SOD and catalase has been observed in primary human leukemia cells [35,36]. In certain cases, the levels of MnSOD in cancer and normal cells are controversial as, for example, in certain types of tumors, lower levels of MnSOD protein and its activity have been found. However, several lines of evidence strongly support the idea that increased antioxidant defenses are engaged by cancer cells to escape severe oxidative damage and to survive in the presence of intrinsic oxidative stress. Accordingly, K-ras transformed cells exhibit enhanced intracellular GSH levels [37]. Similarly, c-myc oncoprotein regulates, at the transcriptional level, γ-glutamyl-cysteine synthetase (γ-GCS), which catalyzes the first rate-limiting step in GSH biosynthesis, and the exposure to H_2_O_2_ increases GSH biosynthesis through the c-myc-γ-GCS axis, thus ensuring enhanced protection against oxidative stress [38]. 

## 4. ROS Exert Several Effects on Cancer Cells

In cancer cells, ROS increase the rate of mutagenicity, which leads to DNA damage and chromosomal instability, thereby potentiating cancer progression [39,40]. High ROS levels lead to a severe increase in chromosome breaks and fragments, end-to-end fusions (dicentrics, Robertsonian-like fusions, rings), and translocations [40]. 

Secondly, ROS may promote cell survival and proliferation, thus contributing to cancer development [41]. ROS can coordinate a variety of redox-sensitive transcription factors, such as NFκB, Nrf2, HIF, and p53 (reviewed in [42]). A moderate increase of ROS often leads to NFκB activation, with subsequent induction of genes encoding for proteins that inhibit apoptosis, including Bcl-xL, cellular inhibitors of apoptosis (cIAPs), FLICE inhibitory protein (FLIP_L_), Gadd45, andTNFR-associated factors (TRAF)-1 and -2 [43]. Nrf2 promotes cell survival mainly through the regulation of several oxidative stress-inducible genes, like those encoding for heme oxygenase-1, ubiquitin/PKC-interacting protein A170, PRDX1, catalase, GPx, SOD, and TRX [44]. HIFs promote cell survival under hypoxia, essentially switching cellular metabolism from mitochondrial respiration to glycolysis. Under basal or low oxidative stress, p53 exerts antioxidant effects, thereby protecting cells from the accumulation of DNA damage and favoring repair mechanisms, and thus allowing cell survival [26]. 

Other than acting at the transcriptional level, ROS can also act at the signal-transduction level to exert pro-survival functions. Oxidative stress can activate the ERK/MEK and the PI3K/AKT pathways. This could result in the inactivation of proapoptotic proteins and in the upregulation of antiapoptotic genes (reviewed in [45]).

Thirdly, ROS can also participate in the metastatic process, by directly stimulating cell invasiveness and cell migration [46]. The ability of transformed cells to invade surrounding tissues strongly depends on matrix metalloproteinases (MMPs), as MMPs degrade the structural components of the extracellular matrix (ECM), and cell-cell and cell-ECM adhesion molecules [47]. Interestingly, MMP-13, -3, and -10, which are involved in the remodeling of collagenous ECM and in the degradation of basement membrane components, are remarkably upregulated by prolonged H_2_O_2_-induced oxidative stress and can contribute to the malignant transformation of cultured epithelial cells [46]. Still, MMP-3 can cause an epithelial-mesenchymal transition, promoting mammary carcinogenesis [48]. NOX1-generated ROS regulate invasion in K-Ras-transformed cells by modulating MMP-9 at both mRNA and protein levels through NFkB signaling [49]. In human pancreatic cancer cells, epidermal growth factor (EGF) induces the secretion and activation of MMP-2 through an intracellular signaling pathway that involves PI3K- and Src-dependent activation of Rac1, which, in turn, is responsible for the NOX-mediated ROS production [50]. In human prostate cancer cells, the expression of ADAM9 metalloproteinase is likely upregulated by NOX1-derived ROS, and could further promote cancer cell invasion and migration [51]. 

In the metastatic growth, the process of new blood vessel formation from pre-existing vessels, namely angiogenesis, also plays an important role. Angiogenesis essentially involves endothelial cells and is regulated by several stimuli (hypoxia, growth factors, cytokines, and oxidative stress), which contribute to the modulation of vascular endothelial growth factor (VEGF), angiopoietin (ANG), EGF, fibroblast growth factor (FGF), transforming growth factor (TGF), platelet-derived growth factor (PDGF), and their receptors (reviewed in [52]). Both *in vitro* and *in vivo* studies report that ROS signaling is crucial for triggering the angiogenic response (reviewed in [53]). In particular, exogenous ROS induced by growth factors may increase neovascularization [54,55]. For instance, H_2_O_2_ is required for the modulation of VEGF expression through the activation of EGF-induced PI3K/AKT/p70S6K1 and insulin-induced MEK/ERK signaling pathways, in ovarian and prostate cancer cells, respectively. Recent studies indicate that even endogenous ROS are involved in regulating angiogenesis and tumor growth. The overexpression of NOX1 increases the tumorigenic potential of NIH3T3 mouse fibroblast and DU-145 human prostate cell lines through H_2_O_2_-induced upregulation of VEGF mRNA and VEGF receptors [56]. Higher H_2_O_2_ levels and increased NOX1 mRNA levels are present in human prostate cancer if compared to those of normal tissue [57]. Similarly, increased NOX4-generated ROS are required for the induction of angiogenesis through HIF-1 and VEGF expression [58]. 

## 5. Excessive ROS Levels Cause Cell Damage and Cell Death

While baseline or, at least, controlled ROS levels exert a prominent pro-survival effect in cancer cells by acting as essential intracellular second messengers for several cytokines and growth factors, high ROS levels can cause cellular damage, and even cell death. Although cancer cells have developed adaptive mechanisms to minimize the effects of oxidative damage, excessive ROS levels can disrupt redox homeostasis, and hence affect death or survival fate, either by irreversibly damaging cellular macromolecules, including DNA, carbohydrates, protein, and lipids (reviewed in [59]), or by modulating redox-sensitive signaling proteins at the levels of signal transduction or transcriptional regulation (reviewed in [42]), or both. 

Proteins can be severely damaged by oxidative stress-induced carbonylation, S-nitrosylation, glutathionylation, and tyrosine nitration. Among protein oxidative modifications, carbonylation is extremely important, as it occurs at high levels *in vivo* and is often used as a marker of oxidative stress. Protein carbonyls are formed either by a direct oxidative attack on the amino acid side chains of proline, arginine, lysine, and threonine, or by an indirect reaction of the primary amino-group of cysteine, lysine, and histidine with reactive carbonyl compounds (ketoamines, ketoaldehydes, deoxyosones) or with carbonyl-containing oxidized lipids [60]. Carbonylation has two main effects. Firstly, heavily carbonylated proteins tend to form high-molecular-weight aggregates that are resistant to degradation. They accumulate within the cell, as damaged or unfolded proteins that can also inhibit proteasome activity [61]. Secondly, carbonylation can severely alter both the structure and function of a variety of proteins. For instance, actin carbonylation causes the disruption of the actin cytoskeleton, leading to drastic cellular function impairments [62]. Still, the inactivation of TRX and TRX reductase, through the carbonylation of their active-site cysteine and selenocysteine residues, is linked to dysregulation of cellular redox status and stress signaling [63]. Moreover, ROS-induced modifications target several other proteins that are important in cell signaling events, including those involved in cell death or survival, *i.e.*, NFκB, AP-1, H-Ras, MAPK, IP3 kinase, PKC-ε, Ras, p53, HIF-1, ASK-1, Bcl-2, caspases, JNK, and p38 MAPK (reviewed in [64]). 

Lipids are also susceptible to oxidative modification. Lipid peroxidation leads to the formation of lipid radicals and several low-molecular-weight decomposition products, such as acrolein, malondialdehyde, and 4-hydroxy-2-nonenal, that are highly reactive towards proteins, DNA, and phospholipids (reviewed in [65]). These aldehydic molecules mediate toxic effects both in cell and animal models. Likewise, oxidized phospholipids activate signaling pathways leading to various inflammatory responses or apoptosis (reviewed in [66]). Through the direct oxidation of cellular macromolecules, ROS induces cell damage and impairs mitochondrial respiration, thus triggering apoptotic cell death.

## 6. Targeting Intrinsic Oxidative Stress in Cancer Cells

The persistent pro-oxidative state characterizing cancer cells, as well as their multiple adaptation mechanisms, can be exploited to develop new therapeutic strategies. In this field, the most remarkable observation is related to the possibility of specifically targeting redox alterations, which are specific for cancer cells, in order to ensure a good therapeutic selectivity. Because the overall redox homeostasis is maintained by the balance between ROS generation and elimination, exogenous compounds that increase ROS generation, or inhibit ROS elimination, can favor the accumulation of ROS in cancer cells, and hence induce cell damage or even cell death [67]. Several agents have been identified that promote ROS generation, *i.e.*, mitochondrial electron transport chain modulators (e.g., arsenic trioxide), redox-cycling compounds (e.g., motexafin gadolinium), or that disrupt antioxidant defenses, *i.e*., GSH depleting agents (e.g., buthionine sulphoximine, phenethyl isothiocyanate), and inhibitors of SOD (e.g., 2-methoxyestradiol), and catalase (e.g., 3-amino-1,2,4-triazole) [67,68,69].

Several studies indicate that arsenic trioxide (ATO), alone, or in combination with other drugs or compounds interfering with ROS metabolism, contributes to the killing cancer cells through the generation of ROS. It directly interferes with the mitochondrial respiration function and determines an increase in O_2_^−^ levels [70]. The effect of ATO has been reported for a large variety of cancer cells, such as human acute promyelocytic leukemia cells [71], A431 epidermoid carcinoma cells [72], A549 lung cancer cells [73], and cervical cancer cells [74]. Motexafin gadolinium (MGd) alters ROS levels by oxidizing many intracellular metabolites [75], and by targeting TRX reductase and ribonucleotide reductase [76]. MGd is undergoing clinical trials, including phase II for the treatment of refractory chronic lymphocytic leukemia, and is also promising for the treatment of brain metastasis. 2-methoxyestradiol (2-ME) induces apoptosis in leukemia cells [8], in ovarian cancer cells [77], and in nasopharyngeal carcinoma cells [78], by inhibiting SOD and by determining the accumulation of superoxide. Together with SOD, catalase also has a central role in the protection of cancer cells against intrinsic oxidative stress. For this reason, catalase inhibitors can contribute to the resensitization of cancer cells to ROS-induced apoptosis [79].

## 7. GSH Depletion in Cancer Cells

Several studies investigated the ability of GSH depleting agents to selectively sensitize cancer cells to overbalanced ROS, so as to cause cell death. GSH is one of the main cellular scavengers of free radicals. GSH depletion may be achieved by inhibiting its synthesis, by accelerating its efflux from mitochondria, or by inducing its conjugation with highly reactive compounds. 

Buthionine sulfoximine (BSO) has been investigated for several years, especially for its capability to prevent *de novo* synthesis of GSH. It is an aminosulfoximine, which inhibits γ-GCS, the rate-limiting step in the synthesis of GSH [80]. BSO exhibits anticancer activity by enhancing oxidative stress-induced citotoxicity and apoptotic cell death in a variety of human cancer cells, *i.e.*, neuroblastoma cells [81], AsPC-1 pancreatic carcinoma cells [82], MCF-7 breast cancer cells [83], and acute promyelocytic leukemia cells [84]. BSO-induced GSH depletion decreases the metastatic growth of murine B16M-F10 cells in the liver, and its combination with antisense Bcl-2 oligodeoxynucleotide completely abolishes metastatic invasion [85]. 

Acivicin also targets GSH synthesis. It is a potent inhibitor of the γ-glutamyl transpeptidase that enhances intracellular GSH synthesis [86] by recycling cysteine within the extracellular pool of GSH. Acivicin, in combination with other GSH-modulating agents, regresses murine metastatic melanoma [87], but its neurotoxicity in humans ruled it out from clinical practice [88]. 

Verapamil can stimulate GSH transport through the multidrug resistance-associated protein (MRP)-1. In combination with antisense Bcl-2 oligodeoxynucleotide, verapamil increases B16M-F10 cell cytotoxicity, thus decreasing their metastatic potential [89]. Together with verapamil, inhibitors of the x_c_^−^ cystine/glutamate antiporter (e.g., sulfasalazine), may also deplete GSH by reducing the uptake of cystine, which is a precursor of cysteine; hence x_c_^−^ cystine/glutamate antiporter inhibitors are also involved in the biosynthetic pathway of GSH [90]. The importance of this antiporter in the growth and progression of cancers has been demonstrated in human pancreatic cancer [91], as well as in small-cell lung cancer (SCLC) cell lines [92]. In SCLC cells, sulfasalazine induced marked growth inhibition, accompanied by GSH depletion [92].

Chloroquine depletes cellular GSH content by increasing levels of nitric oxide and by conjugating with GSH to form an *S*-nitroso-glutathione adduct. Recently, it has been shown that this mechanism causes GSH depletion, with subsequent cell death, in A172 human glioblastoma cells [93]. 

## 8. Flavonoids Interfere with ROS Metabolism

Flavonoids are a large heterogeneous group of benzo-γ-pyrone derivatives, which share a common carbon skeleton of dyphenylpropanes [94], and which can be divided into six different classes, namely flavonols, flavones, flavanones, flavanols, isoflavones, and anthocyanidins, according to their molecular structure [95]. Flavonoids are present in a large variety of foods and beverages, including fruits, vegetables, aromatic plants, medical herbs, tea, and red wine (reviewed in [96]). Results from cell culture and animal models reveal that flavonoids exert preventive effects in carcinogenesis essentially because of their antioxidant activity and their capacity to modulate proteins involved in cell proliferation and cell death pathways. Moreover, in contrast to their activity as antioxidants, certain types of flavonoids act as pro-oxidants, and induce apoptosis in cancer cells, by increasing ROS levels or by modulating detoxifying enzymes, or both. 

For example, kaempferol (3,4',5,7-tetrahydroxyflavone) induces apoptosis in glioma cells by increasing intracellular oxidative stress [97]. Myricetin and quercetin strongly inhibit TRX reductase [98], which exerts several functions in cellular redox control, antioxidant defense, and cell viability. Together with TRX reductase, flavonoids also modulate other detoxifying enzymes, including cycloxygenase [99], inducible nitric oxide synthase [100], monoxygenase, NADH-oxidase, and GSH reductase [101]. They can also alter intracellular GSH content [102]. In particular, 2′-hydroxychalcone, 2′,2-dihydroxychalcones (DHC), 2′,3-DHC, 2′,4-DHC, and 2′,5′-DHC are able to efficiently deplete GSH in A549 lung cancer and HL-60 myeloid cancer cells, whereas chrysin and apigenin are most effective in PC-3 prostate cancer cells [102]. Their anticancer properties are not only linked to the ability to modulate GSH level, but also to the capability to increase the toxicity of known prooxidants (rotenone, 2-methoxyestradiol, and curcumin) [102]. 

## 9. Quercetin Interferes with ROS Metabolism

Quercetin (3,3’,4’,5,7-pentahydroxyflavone, Qu) is an important dietary flavonoid, present in different vegetables, fruits, seeds, nuts, tea, and red wine (reviewed in [103]). Interestingly, Qu can interfere with ROS metabolism and can cause subsequent apoptosis. On the one hand, Qu strongly increases intracellular ROS levels, as Qu radicals (Qu-O•) can be formed after peroxidase-catalyzed oxidation in order to scavenge reactive peroxyl radicals [104]. In certain situations, Qu can generate enough ROS to cause free radical-induced apoptosis through, at least, the ROS/AMPKα1/ASK1/p38 and the AMPKα1/COX2 signaling pathways [105]. Accordingly, the generation of ROS determines the subsequent activations of AMPKα1 and ASK1, which are, in turn, accompanied by p38 activation and recruitment of caspases [106,107]. COX-2 is another AMPKα1 downstream target mediating Qu-induced apoptosis [108]. 

**Figure 1:**
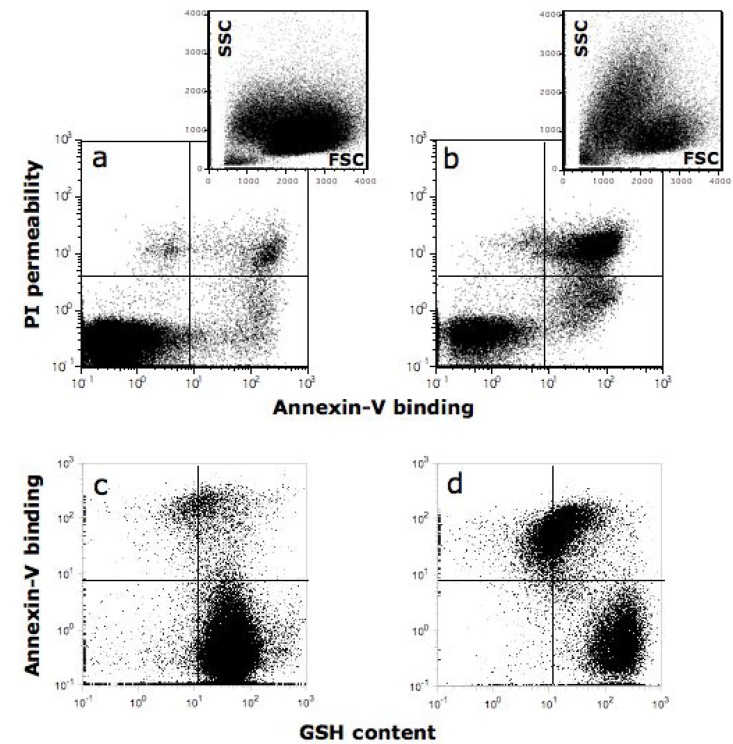
Quercetin induces apoptosis and changes in GSH content in SW872 liposarcoma cells. Polychromatic cytofluorimetric analysis of SW872 cells stained with Alexa 647-labeled annexin-V (ANX-V) and propidium iodide (PI) to detect apoptosis (panels a and b), and with monobromobimane (MBB) to detect intracellular glutathione (GSH) content (panels c and d). Cells were incubated in the absence (left column) or in the presence of 50 μM Qu (right column) for 48 hours. Physical parameters (FSC *vs*. SSC) are shown in the inserts of panels a and b. It is important to note that: (i) the amount of early apoptotic (ANX-V+/PI-) and of late apoptotic cells (ANX-V+/PI+) significantly increases in the presence of Qu (compare panels a and b); (ii) cells that survive the pro-oxidant stress induced by Qu have high GSH content, whereas apoptotic cells have low GSH content (see in panel d that ANX-V negative cells have an increased GSH content, as revealed by MBB fluorescence).

On the other hand, Qu can alter ROS metabolism by directly lowering the intracellular pool of GSH [102,109,110]. By choosing the U937 monoblastic and CEM lymphocytic cell lines as models, we showed that long time exposure to Qu resulted in a decrease in H_2_O_2_ and a reduction in GSH content, suggesting that an inability of Qu to cope with ROS for a long period [111]. By reacting with ROS, Qu can form potentially toxic oxidation products, *i.e.*, semiquinone radical and quinone radicals [112], that are highly reactive toward thiols; these radicals thus preferentially react with GSH [113]. Qu depletes GSH in a concentration-dependent manner; the higher the Qu concentration, the more GSH is depleted, presumably because GSH reacts with Qu-derived semiquinone and quinine radicals. In a model system of isolated rat liver nuclei, Qu reduces, in a dose-dependent manner, both the nuclear GSH content and the glutathione S-transferase activity [114]. Then, in the presence of Qu-induced GSH depletion, apoptosis is triggered trough mitochondrial depolarization (Figure 1), as described in several models [115,116,117,118,119,120]. In particular, during Qu-induced apoptosis, loss of mitochondrial membrane potential, phosphatidilserine exposure, and decrease of mitochondrial mass are early events that precede permeability to propidium iodide and loss of DNA [117,121]. GSH depletion is also responsible for the efficacy of the Qu-arsenic trioxide combination therapy in U937 cells [110]. Considering that the detoxification of arsenic trioxide essentially depends on glutathione S-transferases, and that arsenic trioxide is highly reactive towards thiols [122], intracellular GSH depletion may result in the increase of intracellular free arsenic trioxide concentration, and hence, increase protein damage. 

Intriguingly, a recent study reports that the natural compound β-phenylethyl isothiocyanate (PEITC) causes massive ROS accumulation in Ras-transformed cells by depleting GSH and inhibiting GPx enzyme activity. The compound disables the GSH antioxidant system at two different steps [123]. Moreover, PEITC can effectively eliminate fludarabine-resistant chronic lymphocytic leukemia cells [124], as well as Gleevec-resistant chronic myelogenous leukemia cells [125], through a redox-mediated mechanism with low toxicity to normal lymphocytes.

## 10. Quercetin Induces Apoptosis in Cancer Cells

The study of Qu as potential chemopreventers, or for chemotherapy, is assuming increasing importance, considering its high toxicity for cancer cells, along with its relatively scarce effects on normal, non-transformed cells [126]. Cancer cells are extremely sensitive to Qu-induced apoptosis. These differences between normal and cancerous cells may result from different signaling pathways [117,127,128,129,130,131,132,133]. 

Qu triggers apoptosis via the mitochondrial pathway, through the loss of mitochondrial membrane potential, the subsequent release of cytochrome *c* from mitochondria to cytosol, and the final activation of caspases, such as caspase-3 and caspase-7 [127,134,135]. Qu also modulates pro-apoptotic and anti-apoptotic proteins. In particular, Qu induces the upregulation of Bax and Bak, and the downregulation of Bcl-2 and Bcl-xL, thus determining the multimerization of Bax to the mitochondrial membrane. These events are then accompanied by the cleavage of procaspase 9 and of poly(ADP-ribose) polymerase (PARP) [134,135,136]. Quercetin also inhibits the PI3K/Akt pathway, which regulates cell survival and proliferation, by decreasing enzymatic PI3K activity without affecting p85 or p110 subunit levels [136,137]. Qu-induced apoptosis may occur also via the death-receptor-dependent pathway. In prostate cancer and hepatoma cells, Qu is responsible for the upregulation of death receptor (DR)-5, which belongs to the TNF family, and which is activated by TNF-related apoptosis-inducing ligand (TRAIL) [129,130]. The downregulation of c-FLIP (an inhibitor of caspase-8) and the upregulation of DR5 participate in the increased sensitization of hepatoma cells to Qu-induced apoptosis *via* TRAIL [130]. 

Several studies suggest that survivin plays an important role in Qu-induced apoptosis, even if its precise regulation still remains unclear. The connection between the downregulation of survivin (determined by Qu) and apoptosis has been reported for several cancer cell lines, including HepG2 [138], SW480 colon carcinoma [132,133], glioma [139], and non-small cell lung cancer [140]. Owing that survivin is a strong caspase inhibitor, its suppression, either by downregulation of the putative mRNA, or by promoting its degradation by the proteasome, can contribute to cancer cell death [141]. Furthermore, in certain cases, the Qu pro-apoptotic effect is mediated by the downregulation of heat shock protein (Hsp)-90, in a caspase-dependent mechanism [142]. Likewise, the Qu derivative, phenylisocyanate of quercetin (PHICNQ), induces apoptosis by downregulating Hsp-70 in human K562 chronic myeloid leukemia cell lines [143]. As indicated above, Qu induces apoptosis through ROS generation and via the subsequent activation of the ROS/AMPKα1/ASK1/p38 and the AMPKα1/COX2 signaling pathways [105,108].

Conversely, non-transformed cells are less sensitive to Qu-induced cell death, as well as to several different GSH-depleting stimuli [144,145]. Recently, we have shown that treating human peripheral blood lymphocytes (PBL) with Qu causes a loss of mitochondrial membrane potential only in a small amount of cells, and that this effect occurs only at concentrations (>100 µM) that are definitely high if compared to concentrations that are able to induce cell cycle arrest, and even apoptosis, in cancer cells. In tumor cells, Qu concentrations ranging from 3.5 μM to 25 μM are sufficient to exert antiproliferative and proapoptotic effects [104,146,147]. Moreover, in activated or proliferating PBL, cell death seemed to be independent from the entrance into the cell cycle of resting lymphocytes (a phenomenon that typically occurs after antigenic stimulation). In this model, Qu did not even increase PBL susceptibility to CD95-induced apoptosis [49,126].

## 11. Relevance of Quercetin for Human Health

The chemopreventive properties of Qu have been studied for a long time. Several *in vivo* studies on animal models report its efficacy in the prevention of different types of cancer induced by potent carcinogens, such as benzo(a)pyrene, azoxymethane, and *N*-nitrosodiethylamine [148,149,150]. The anticancer capability of Qu has also been demonstrated in animal models [146,151]. 

More extensive investigations in humans are needed, as only a few clinical studies have evaluated Qu as a potential chemotherapeutic. The first investigation determined the pharmacokinetics of a water-soluble pro-drug form of Qu (QC12), but did not evaluate any clinical effects [152]. The more recent second clinical study demonstrates that the combination of curcumin, which acts on mitochondria [153], and Qu reduces the number and size of adenomas in patients with familial adenomatous polyposis [154]. Still, the combination chemotherapy of tamoxifen and quercetin exerts anticancer effects through the inhibition of angiogenesis [155]. 

As far as safety studies are concerned, the almost total lack of adverse effects in preclinical studies (in terms of acute toxicity, and *in vitro* and *in vivo* carcinogenicity), at least at the estimated dietary intake, renders Qu an interesting candidate for clinical applications in the treatment of certain forms of cancer (reviewed in [156]). Nevertheless, it is important to highlight that the ability of Qu to directly poison topoisomerase II (topo-II), through the stabilization of double strand breaks in the topo-II-DNA cleavage complexes, could account for genetic rearrangements leading primary hematopoietic progenitor cells that develop into mixed-lineage leukemia. This means that more studies are needed to assess the range of Qu concentrations that can exert a biological anticancer effect without affecting human health.

**Figure 2:**
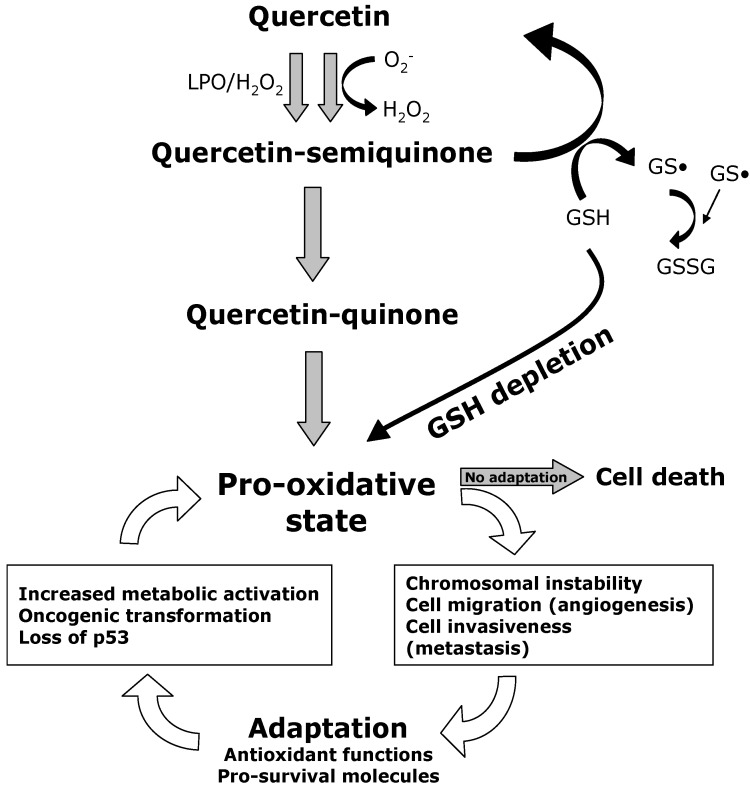
The role of quercetin in the metabolism of ROS in cancer cells. Reactive oxygen species (ROS) are continuously generated in cancer cells as a result of several factors, including increased metabolic activity, the activation of oncogenes, and the eventual loss of p53. The increase of ROS plays an important role in the maintenance of cancer phenotype and leads to a condition of pro-oxidative state. In turn, ROS can determine chromosomal instability (accumulation of mutations and deletions), and can stimulate cell growth and proliferation, as well as cell migration and invasiveness (angiogenesis and metastasis). The adaptation of cancer cells to this setting essentially involves the rearrangement of the antioxidant functions and the upregulation of pro-survival proteins. These changes allow them to bypass the cell death caused by excessive levels of ROS. The exposure to Quercetin (Qu) leads to the formations of quercetin-semiquinones and quercetin-quinones, which exert pro-oxidant effects within the cells. These compounds are highly reactive towards thiols and react with reduced glutathione (GSH), causing GSH depletion. The disruption of GSH antioxidant defense in cells with persistent ROS overload, like malignant cells, leads to cell death by apoptosis. LPO, lactic peroxidase; O_2_^−^, superoxide anion; H_2_O_2_, hydrogen peroxide; GSH, glutathione; GS•, oxidized GSH; GSSG, glutathione disulfide.

## 12. Concluding Remarks

The capability of Qu to act as a pro-oxidant and to induce GSH depletion in cancer cells definitely represents a fascinating tool in the field of oncology. The use of Qu to treat cancer cells could contribute to the abrogation of the adaptive mechanism that ensures the maintenance of redox homeostasis under persistent endogenous oxidative stress (Figure 2). Owing to the biological and biochemical differences between cancerous and normal cells, the targeting of ROS metabolism could help bypass drug resistance and achieve selectivity of treatment, while maintaining a weak or null impact on normal cells. Because normal, non-transformed cells have a lower basal intracellular ROS level, and have a full antioxidant capacity, they should be less vulnerable to the ROS stress that is induced by Qu. In the continuous effort to identify chemotherapeutic drugs that selectively kill tumor cells without side effects on normal cells, Qu certainly remains an attractive compound, even if further studies are needed. 
